# Boosting large‐scale river connectivity restoration by planning for the presence of unrecorded barriers

**DOI:** 10.1111/cobi.14093

**Published:** 2023-05-01

**Authors:** Christina T. Ioannidou, Thomas M. Neeson, Jesse R. O'Hanley

**Affiliations:** ^1^ Kent Business School University of Kent Canterbury UK; ^2^ Department of Geography and Environmental Sustainability University of Oklahoma Norman Oklahoma USA; ^3^ Durrell Institute of Conservation and Ecology University of Kent Canterbury UK

**Keywords:** habitat restoration, hidden barriers, missing data, optimization, river connectivity, barreras ocultas, conectividad de ríos, datos faltantes, optimización, restauración del hábitat, 栖息地恢复, 河流连接度, 缺失数据, 隐性障碍, 优化

## Abstract

Conservation decisions are invariably made with incomplete data on species’ distributions, habitats, and threats, but frameworks for allocating conservation investments rarely account for missing data. We examined how explicit consideration of missing data can boost return on investment in ecosystem restoration, focusing on the challenge of restoring aquatic ecosystem connectivity by removing dams and road crossings from rivers. A novel way of integrating the presence of unmapped barriers into a barrier optimization model was developed and applied to the U.S. state of Maine to maximize expected habitat gain for migratory fish. Failing to account for unmapped barriers during prioritization led to nearly 50% lower habitat gain than was anticipated using a conventional barrier optimization approach. Explicitly acknowledging that data are incomplete during project selection, however, boosted expected habitat gains by 20–273% on average, depending on the true number of unmapped barriers. Importantly, these gains occurred without additional data. Simply acknowledging that some barriers were unmapped, regardless of their precise number and location, improved conservation outcomes. Given incomplete data on ecosystems worldwide, our results demonstrate the value of accounting for data shortcomings during project selection.

## Abstract (es)

Incremento de la restauración de la conectividad a gran escala de los ríos mediantze la planeación de la presencia de barreras sin registro

**Resumen:**

Las decisiones de conservación se toman con datos incompletos de la distribución, hábitat y amenazas de las especies, pero los marcos para asignar fondos de conservación rara veces lo consideran. Analizamos cómo la consideración explícita de los datos faltantes puede incrementar la rentabilidad de la inversión en la restauración de ecosistemas. Nos enfocamos en el reto que es la restauración de la conectividad entre ecosistemas acuáticos mediante la eliminación de presas y cruces de carreteras en los ríos. Desarrollamos y aplicamos una forma novedosa de integrar la presencia de las barreras sin registro dentro de un modelo de optimización de barreras en el estado de Maine (Estados Unidos) para maximizar la ganancia esperada de hábitat para los peces migratorios. La omisión de las barreras sin registro durante la priorización resultó en una ganancia de hábitat casi 50% menor a la anticipada cuando se usó una estrategia convencional de optimización de barreras. Sin embargo, el reconocimiento explícito de los datos incompletos durante la selección del proyecto incrementó la ganancia esperada de hábitat en un promedio del 20–273%, dependiendo del número real de barreras sin registro. Estas ganancias ocurrieron sin datos adicionales. Los resultados de conservación aumentaron con tan sólo el reconocimiento de que algunas barreras no estaban registradas, sin importar el número y ubicación precisos, Ya que hay datos incompletos para todos los ecosistemas a nivel mundial, nuestros resultados demuestran lo importante que es considerar la carencia de datos durante la selección de proyectos.

## 对未记录障碍物的存在进行规划以促进大尺度河流连接度的恢复

【摘要】保护决策经常是在物种分布、栖息地和威胁数据不完整的情况下制定的, 但分配保护投资的框架却很少考虑数据缺失的问题。本研究探讨了明确考虑缺失数据的方法如何提高生态系统恢复的投资回报, 重点关注通过移除河流中的水坝和交叉路口来恢复水生生态系统的连接度。我们开发了一种新的方法, 将未测绘障碍物的存在纳入障碍物优化模型, 并将该模型用于美国缅因州来帮助洄游鱼类获得最大程度的预期栖息地增加。我们发现, 如果在确定优先保护时不考虑未绘制的障碍物, 会导致栖息地的增加比传统的障碍物优化方法得到的预期低近 50%。然而, 在项目选择过程中明确认定数据的不完整性, 则可以使预期栖息地增加平均提高 20‐273%, 具体数值取决于未测绘障碍物的真实数量。这些增加的栖息地都是在没有额外数据的情况下得到的。结果表明, 仅仅将未测绘障碍物纳入考虑, 不论其确切数量和位置如何, 都能改善保护结果。鉴于全球生态系统普遍的数据不完整性, 本研究的结果证明了在项目选择过程中考虑数据缺失的价值。**【翻译:胡怡思;审校:聂永刚】**

## INTRODUCTION

Conservation actors worldwide make substantial investments in restoration projects to conserve biodiversity and ecosystem services, mitigate threats, restore habitats, and enhance ecosystem connectivity. Often, conservation projects are selected using return‐on‐investment (ROI) frameworks that aim at maximizing conservation benefit per dollar spent. ROI frameworks have been used, implicitly or explicitly, to direct spending for terrestrial and marine reserve networks (Church et al., 1996; Meester et al., 2004), habitat restoration (Orsi et al., 2011), restoration of connections among habitat patches (Williams, 1998), payments for ecosystem services (Hu et al., 2015), and a range of other conservation actions. When ROI frameworks are used implicitly, decision makers rely on expert judgement to identify a set of cost‐effective projects. When done explicitly, resource allocation decisions are based on quantitative approaches that seek to identify an optimal budget allocation among candidate projects.

An ROI framework can be an effective tool for allocating resources, but a tacit assumption of most frameworks is that underlying data on species’ distributions, habitats, and threats are complete or at least unbiased and sufficient. Although conservation actors certainly recognize that data are imperfect (Possingham et al., 2007), the implications of data shortcomings are rarely acknowledged during planning (Boitani et al., 2011). Failure to explicitly acknowledge incomplete data during planning can potentially lead to an overestimation of conservation benefits or an underestimation of costs and impacts of proposed projects. Of greater concern are instances where better data would have changed the priority ranking of candidate projects and led to an alternative allocation of conservation resources (Kujala et al., 2018).

We addressed a fundamental question: How might ROI frameworks be modified to account for the fact that conservation decisions are necessarily made with incomplete data? Decision makers in other domains deal with uncertainty and missing data by applying flexible decision frameworks under the presumption that decisions are reversible and flexible (Fovargue et al., 2021). Many conservation decisions, however, require significant investment outlay that is not easily reversible, for example, the financial and sociopolitical costs of establishing protected areas, large capital costs of many restoration projects (e.g., dam removal or cleanup of superfund sites), and opportunity costs associated with failing to prevent a species’ impending extinction. High extinction rates and rapid loss of ecosystem services also preclude a wait‐and‐see strategy of taking no action while more data are acquired (Grantham et al., 2009). In many cases, it is not cost‐effective to acquire more data (Grantham et al., 2008; Hermoso et al., 2013)

To explore how ROI frameworks might be modified to account for incomplete data, we focused on the challenge of restoring aquatic ecosystem connectivity by removing dams and impassable road crossings from rivers. Landscape connectivity is crucial for biological conservation (Fahrig, 2003; Fischer & Lindenmayer, 2007; Lucas & Baras, 2001), and rivers are particularly vulnerable to barrier fragmentation due to their dendritic structure (Kemp & O'Hanley, 2010). In the United States alone, there are an estimated 78,000 dams over 3 m tall and as many as 3–8 million smaller structures that affect river flow (Doyle & Havlick, 2009). This is highly likely to be a vast undercount based on recent findings from Europe (Belletti et al., 2020) and the United States (Buchanan et al., 2022).

Restoration of river connectivity through dam removal and other barrier mitigation actions is an integral strategy for improving freshwater ecosystems (Bednarek, 2001; Roni et al., 2002). Millions of dollars are spent annually in the United States alone on connectivity restoration (Bernhardt et al., 2005; Roni et al., 2008), with planning carried out by a host of stakeholders (e.g., local watershed councils, national nongovernmental organizations, state and federal agencies) focused on a wide range of spatial scales, from small watersheds (O'Hanley, 2016), to basins (CBCP, 2022), to entire states (CFPF, 2022), or even to transnational regions (Moody et al., 2017).

Various methods have been suggested for prioritizing river barrier removal and mitigation decisions. Most prioritization approaches focus on enhancing dispersal of migratory fish populations (Ioannidou & O'Hanley, 2018; Kuby et al., 2005; Neeson et al., 2015; O'Hanley & Tomberlin, 2005; Paulsen & Wernstedt, 1995); only a handful of studies concentrate on the dispersal of resident fish (Diebel et al., 2015; O'Hanley, 2011; O'Hanley, Wright, et al., 2013). None of these studies handle uncertainty about the number or location of barriers. In practice though, barrier inventories are far from complete, which these modeling frameworks fail to recognize. In Oregon (USA), for example, around 8900 structures were officially recorded as of 2004. This number subsequently grew to over 28,000 by 2011 and over 40,000 in 2019 (ODFW, 2020). The potential presence of unrecorded or hidden barriers raises a key question: What impact does this have on the effectiveness of large‐scale connectivity restoration?

**FIGURE 1:**
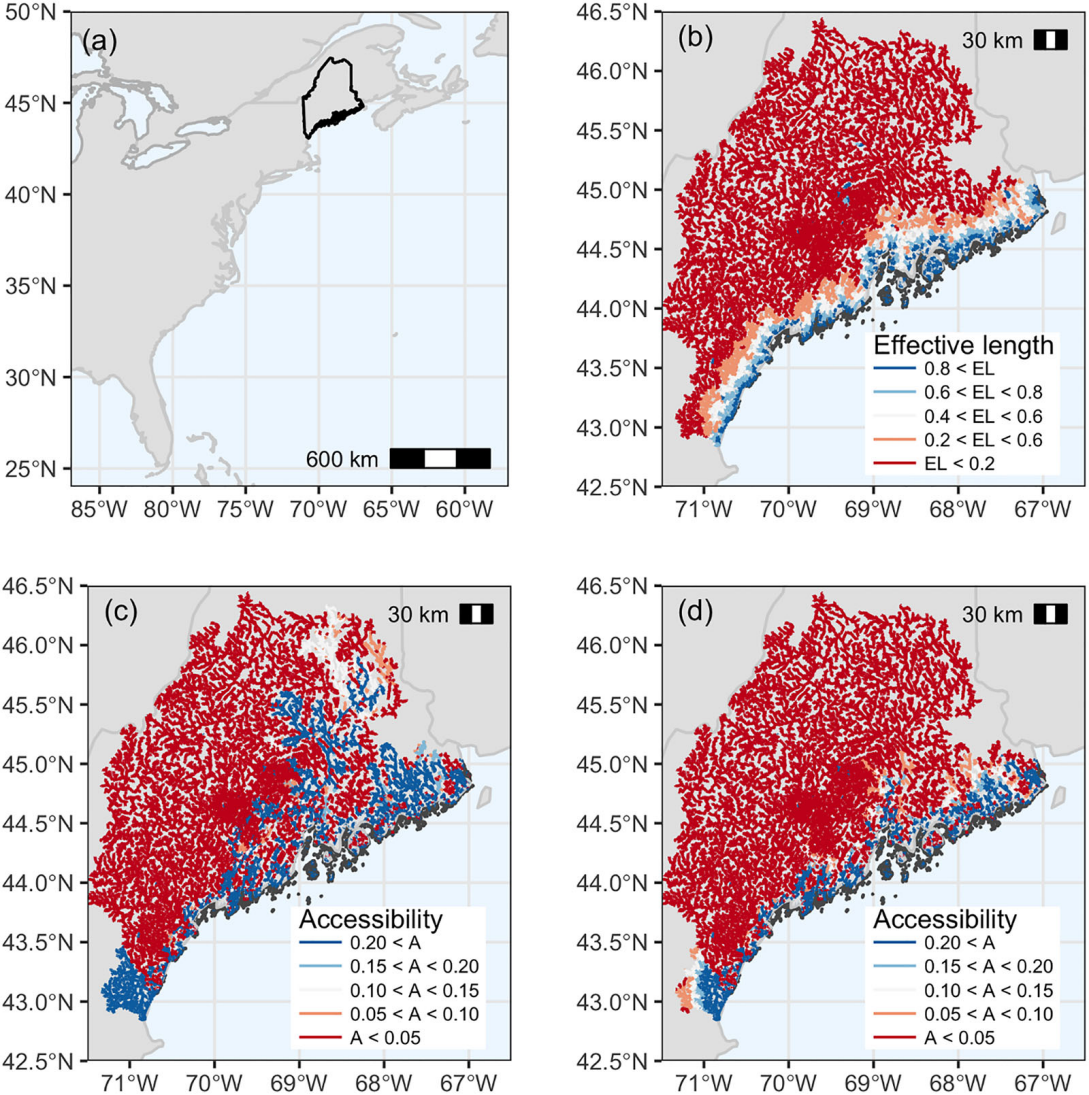
(a) Location of study region (Maine, U.S.A.), (b) fraction of accessible river (i.e., effective length multiplier) assuming 4250 unmapped barriers, (c) cumulative passability (also known as accessibility, given recorded river barriers only), and (d) expected accessibility considering given recorded and 4250 unmapped barriers (i.e., product of cumulative passability and effective length multiplier). EL, effective length multiplier; A, accessibility.

To help answer this question, we developed a novel optimization‐based approach for identifying a portfolio of cost‐effective barrier mitigation projects that considers how hidden (i.e., unmapped) barriers might constrain habitat gains of selected projects. As a case study, we applied this model to the U.S. state of Maine (Figure 1a), where more than US$1 million is invested annually in barrier removals to restore habitat access for endangered Atlantic salmon (*Salmo salar*) (MFWCO, 2023; Hall, 2020) and other fish species. First, we used a naïve version of our model (i.e., one that did not account for hidden barriers) to quantify habitat gains that might be achieved without accounting for data shortcomings. Using this as a baseline, we then quantified increases in ROI that may occur as well as how mitigation choices differ spatially as a result of taking missing barriers into account during project selection. Finally, we performed a sensitivity analysis to quantify how increased ROI depends on the true number of unmapped barriers. In doing so, we sought to demonstrate how accounting for data shortcomings during project selection might improve returns on conservation investments.

## METHODS

### Naïve barrier optimization model

Before proposing our approach for optimizing barrier mitigation given the presence of hidden barriers, we introduce a deterministic model, originally proposed by O'Hanley and Tomberlin (2005) and later extended by King and O'Hanley (2016), to optimize upstream habitat gains from barrier repair and removal assuming all barrier locations are known (hereafter naïve model). It is assumed the river network is strictly dendritic, meaning it never diverges in the downstream direction. This assumption implies there is a unique path from the river mouth to any point upstream. It is also assumed that each barrier can be assigned a numeric passability value corresponding to the fraction of fish (range: 0–1) that can successfully navigate a barrier in the upstream and or downstream direction (Kemp & O'Hanley, 2010). Cumulative passability, in turn, quantifies the combined effect that barriers have on fish as they migrate from the river mouth to areas above barriers (or vice versa) and, assuming passabilities are independent, is evaluated for each barrier by multiplying its passability with the passabilities of all barriers downstream.

We used the following notation: *J*, indexed by *j* and *k*, is the set of all known artificial and natural barriers. Included in *J* is 1 or more dummy barriers located at the mouth of each river outlet. Dummy barriers have passability equal to 1 and are included to account for available habitat situated between the river mouth and the first set of artificial or natural barriers. Term $v_{j}$ expresses the net amount of habitat in river subnetwork *j* (i.e., the section of river immediately above barrier *j* up to the next set of barriers or the river terminus). The set of mitigation projects available at barrier *j* (possibly empty) is represented by $A_{j}$ and indexed by *i*. The cost of implementing project *i* at barrier *j* is given by $c_{ji}$, and the available budget for carrying out mitigation is given by *b*. Set $D_{j}$ is the subset of known barriers downstream from and including barrier *j*. The initial passability of barrier *j* is denoted by $p_{j}^{0}$, and $p_{ji}$ denotes the increase in passability at barrier *j* given implementation of mitigation project *i*. Finally, the decision variables of the model are defined below:

$$x_{ji}=\left\{\begin{array}{cc} 1, & \text{if mitigation project}\;i\;\text{is carried out at barrier}\;j,\\ 0, & \mathrm{otherwise}, \end{array}\right.$$

and

$$\begin{array}{ccc} z_{j} & = & \text{cumulative passability}\;\left(\text{aka accessibility}\right)\;\text{to habitat area}\\ & & \text{immediate above barrier}\;j. \end{array}$$

With this in place, a mathematical formulation of the naïve barrier optimization model is as follows:

(1) $$\max V=\sum_{j\in J}v_{j}z_{j}$$

s.t.

(2) $$\sum_{j\in J}\sum_{i\in A_{j}}c_{ji}x_{ji}\leq b,$$

(3) $$\sum_{i\in A_{j}}x_{ji}\leq 1\;\;\;\;\;\;\;\;\;\;\;\;\;\;\;\;\;\;\;\;\;\;\;\;\;\;\;\;\;\;\;\forall j\in J,$$

(4) $$z_{j}=\prod_{k\in D_{j}}\left(p_{k}^{0}+\sum_{i\in A_{k}}p_{ki}x_{ki}\right)\;\;\;\;\;\;\;\;\;\;\forall j\in J,$$

(5) $$x_{ji}\in \left\{0,1\right\}\;\;\;\;\;\;\;\;\;\;\;\;\;\;\;\;\;\;\;\;\;\;\;\;\;\;\;\;\;\;\;\forall j\in J,i\in A_{j}.$$

The objective function (1) maximizes total accessible habitat *V* by summing cumulative passability‐weighted habitat across all barriers. The inequality (2) places a budget on the total cost of barrier mitigation. Constraints (3) specify that at most 1 mitigation project can be implemented at each artificial barrier *j*. Equations (4) determine the cumulative passability of each barrier *j* by taking the product of barrier passabilities in set $D_{j}$. Passability for any barrier *k* in $D_{j}$ is determined by taking initial passability $p_{k}^{0}$ and adding to it the increase in passability $p_{ki}$ if mitigation project *i* is selected. Equations (4) are nonlinear but can be expressed in linear form using the probability chain technique proposed by O'Hanley, Scaparra, et al. (2013) and explained in King and O'Hanley (2016). Finally, constraints (5) require barrier mitigation decision variables to be binary.

### Informed barrier optimization model

To formulate a model that accounts for the presence of hidden barriers (hereafter informed model), we replaced the objective function (1) with

(6) $$\max\tilde{V}=\sum_{j\in J}{\tilde{v}}_{j}z_{j},$$

where ${\tilde{v}}_{j}$ is the expected net habitat above barrier *j* taking into consideration the presence of hidden barriers downstream from *j* and immediately above *j* (up to the next set of barriers or the river terminus).

In what follows, we assumed the river network can be decomposed into a set of nonoverlapping river segments, denoted by *S* (Appendix S1). The amount of habitat in any given segment *s* is taken to be uniformly distributed along its length. For simplicity and ease of explanation, we used river length as a proxy for habitat, meaning that segments can be readily delineated by all known barriers and confluence points; a more general approach would require first splitting the river network by habitat areas, then by known barriers and confluence points. Accordingly, expected net habitat above barrier *j* is evaluated as

(7) $${\tilde{v}}_{j}=\sum_{s\in U_{j}}{\tilde{l}}_{s},$$

where $U_{j}$ is the subset of segments in river subnetwork *j* (Appendix S1) and ${\tilde{l}}_{s}$ is the effective length of segment *s* due to the presence of hidden barriers.

To calculate effective segment length ${\tilde{l}}_{s}$, we first introduce the following notation. Let $l_{s}$ be the length of segment *s*. The total length of the river network is denoted by $L=\sum_{s\in S}l_{s}$. Further, let $l_{s}^{\prime }$ be the total length of river downstream from segment *s* and let 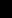 be the total length of river not downstream from or within segment *s*. The total number of hidden barriers in the river network is *n*. We assume their passabilities are independent and identically distributed with mean $\tilde{p}$.

If hidden barriers are randomly located across the river network and the likelihood of hidden barriers being present along segments is proportional to length, it follows that the probability $\pi_{skt}$ (that *k* hidden barriers are located on river segment *s*, *t* hidden barriers are located downstream of *s*, and the remaining n−k−t hidden barriers are located elsewhere in the river network) is characterized by a multinomial distribution with counts *n*, *t*, and n−k−t and event probabilities $\frac{l_{s}}{L}$, $\frac{l_{s}^{\prime }}{L}$, and 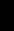. This yields the following formula for $\pi_{skt}$:

(8)

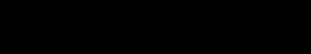




In the case where hidden barriers are not randomly located over the river network, one needs to modify the event probabilities in the above equation (Appendix S2).

Given *t* hidden barriers downstream of segment *s*, it also follows that the conditional expected cumulative passability $E(P_{s}|t)$ of segment *s*, with random variable $P_{s}$ denoting the true (albeit unknown) cumulative passability of *s*, is

(9) $$E\left(P_{s}|t\right)\;={\tilde{p}}^{t}.$$

Meanwhile, it is possible to show (Appendix S3) that the *k* hidden barriers on segment *s* are uniformly distributed lengthwise, which is equivalent to the *k* hidden barriers being on average equally spaced with separation $\frac{\ell_{s}}{k+1}$ between them. Accordingly, the conditional expected length $E(L_{s}|k)$ of segment *s*, with random variable $L_{s}$ denoting the true effective length of *s*, is given by

(10) $$E\left(L_{s}|k\right)=\sum_{r=0}^{k}{\tilde{p}}^{r}\left(\frac{l_{s}}{k+1}\right).$$

For clarity, we provide an example of how conditional expected length (Equation 10) is evaluated for a chosen segment in a hypothetical river network given a varying number of hidden barriers (Appendix S1).

Putting everything together, an exact formula for calculating effective segment length ${\tilde{l}}_{s}$ (assuming independence between hidden barrier passability and distance between hidden barriers) is given by

(11) $${\tilde{l}}_{s}=\sum_{k=0}^{n}\sum_{t=0}^{n-k}\pi_{skt}\cdot E\left(P_{s}|t\right)\cdot E\left(L_{s}|k\right).$$

For very large river networks involving hundreds of thousands of river segments, the procedure outlined above for estimating effective segment lengths ${\tilde{l}}_{s}$ can be computationally taxing. As an alternative, we developed a log‐linear regression model to derive a multiplier for effective segment length (described below).

We emphasize that the only information added to the informed model (compared with the naïve model) is estimates of the number and passability of hidden barriers in the planning area. No additional collection of field data is required. We further stress that the benefits provided from using an informed approach will generally be context dependent. Nonetheless, even a hypothetical application of the model demonstrates that optimal mitigation decisions do indeed change and significant habitat gains can be achieved when even a single hidden barrier with moderate impassability is present in a river network (Appendix S4).

### Maine barrier data set

Georeferenced data on dams, road crossings, and natural barriers throughout Maine (*n* = 26,806) were obtained from the U.S. Fish and Wildlife Service, Gulf of Maine Coastal Program (GMCP). Each recorded structure in the most recent 2018 version of the database includes a description of its structural type (e.g., dam, culvert, multiple culvert, ford, bridge, natural fall), a qualitative assessment of passability (i.e., barrier, potential barrier, no barrier, or unknown), basic physical information like bank‐full width for road crossings and structure height for dams, and, in the case of dams, whether a fish pass has been installed (see Appendix S6 for a complete list of recorded and estimated barrier attributes). For our analysis, we first removed all structures with passability classed as no barrier on the basis that such structures are unlikely to present an obstacle to fish passage. For road crossings, we further removed any structure designated as removed crossing or no crossing given such structures have either already been removed or are nonexistent (i.e., stream and road layer intersections identified by a desktop analysis and initially included in the barrier database but subsequently found to be not present following field surveys). For natural barriers, we also excluded debris jams and beaver dams on the assumption that these are transient features of the landscape and unlikely to impede fish dispersal in the long term.

The remaining subset of structures (*n* = 18,656) was subsequently snapped to a single‐threaded river network derived from the 1:100,000‐scale National Hydrology Dataset Plus with a 100‐m snapping distance. As a final step, we excluded barriers not located in coastal watersheds of Maine. This was required because we did not have access to barrier and stream data for Canada and without this, the connectivity status of rivers and the benefits of barrier mitigation cannot be quantified. The final data set (*n* = 14,902) included 13,913 road crossings, 829 dams, and 160 natural barriers. The river network was subsequently split at each barrier point and net upstream river length above each barrier was determined by matching each river segment to its immediate downstream barrier. The various geospatial data processing steps were performed in ArcGIS 10.3 (ESRI, 2014) with the Barrier Analysis Tool (BAT) add‐in (Hornby, 2013). Finally, we determined Strahler order with the RivEX toolbox for ArcGIS (Hornby, 2014).

Initial barrier passability estimates for recorded barriers were assigned as follows. For road crossings (e.g., culverts, fords, and bridges), we assigned 0.0 passability to structures classed as a barrier and 0.5 to those classed a potential barrier. For crossings classed as unknown, we determined the mean passability of existing crossings (0.47) in the full database, excluding any removed or no crossing structures. We assumed a passability of 1.0 for structures classed as no barrier. For natural falls, we used the same approach, assigning 0.0 to a barrier and 0.5 to a potential barrier. For dams, we assigned an initial passability of 0.0, unless fitted with a fish ladder or fish lift (also known as elevator). The passability of a fish ladder was derived from analysis of field data reported in Bunt et al. (2016). More specifically, we estimated mean attraction (percentage of fish attracted to a fishway entrance) and separately mean passage (percentage of fish successfully exiting a fishway) of Denil‐type fish ladders, excluding a single outlier with very low efficiency (0.21) and no passage (0.0). The product of mean attraction (0.69) and mean passage (0.59) was used as an estimate for overall passability (0.41). The passability of fish lifts (0.66) was estimated in a similar manner by taking the attraction efficiency of a fish ladder and using a high value for passage (0.95) based on expert advice (B. Lake and B. Towler, personal communication) that once fish are trapped, the passage efficiency of a lift approaches 100%.

We considered a single mitigation option for each artificial barrier; natural falls were considered natural features for which mitigation is normally proscribed. For crossings, mitigation was assumed to restore full passability (1.0) and consisted of replacement with an open‐bottom arched culvert for streams with bank‐full width <18.3 m (60 feet) or with a bridge for bank‐full widths 18.3 m (60 feet) or greater. To estimate the cost of installing a new culvert as a function of stream width (the main determinant of cost), we generated a lookup table with 24 different intervals for bank‐full width. Costs, varying from a low of $24,100 to a high of $808,000, were derived from prior analysis (in 2009) performed by the GMCP on data collected by the Salmon Habitat and River Enhancement Project and American Rivers. All monetary units are in U.S. dollars. For our purposes, we took the original cost table produced by GMCP, adjusted the cost figures for inflation (to 2020 U.S. dollars), and extrapolated to stream widths up to 18.3 m (60 feet).

Although the cost of constructing a bridge can vary widely and depends on a number of site‐specific factors, for simplicity we assumed a median cost of $1 million. For small‐sized dams 3.0 m (10 feet) high or less, removal was considered the most cost‐effective mitigation option for restoring full passage (1.0). Inflation‐adjusted costs for removing dams (to 2020 U.S. dollars) were obtained from Graber (2011), with dams 0.6–1.5 m (2–5 feet) high costing $47,600 to remove and dams 1.8–3.0 m (6–10 feet) high costing $86,600 to remove. For medium‐sized dams over 3 m (10 feet) but not exceeding 15.2 m (50 feet), installation of a Denil fish ladder was the preferred option at a cost of $100,000 per vertical foot (B. Lake, personal communication). Passability of a fish ladder was assumed to be 0.41 (same as existing fish ladders). For large‐sized dams in excess of 15.2 m (50 feet) but not exceeding 30.5 m (100 feet), we assumed a fish lift could be installed with passability equal to 0.66 (same as existing fish lifts). The cost of constructing a fish lift varies based on height and river size. More fish typically migrate through large rivers, necessitating more or larger hoppers (B. Lake, personal communication). As a proxy for river size, we used Strahler order and assumed that lifts for large dams <22.9 m (75 feet) cost $10 million for rivers of order 1–5 and $25 million for rivers of order 6 or higher. A 50% increase in cost was added to large dams with height >22.9 (75 feet) (i.e., $15 million for order 1–5 and $37.5 for order ≥6). Dams 30.5 m (100 feet) and over, of which there are 4 in Maine, were not considered candidates for mitigation due to the significant engineering difficulties involved and high cost of constructing lifts on very large dams. The only viable alternative for such dams is trap and haul (also known as trap and truck), but this is not normally considered a long‐term solution (M. Brown, personal communication). For any crossing or dam with a missing record for bank‐full width or structure height, we used median bank‐full width and structure height, respectively, according to stream order.

We anticipated the number of unmapped barriers in coastal watersheds was 1875–7490, with a most likely figure of 3745 (A. Abbott, personal communication). To explore scenarios across this range, we assumed the number of unrecorded barriers is approximated by a triangular distribution parameterized by a lower bound, upper bound, and mode. We determined the minimum, 25th percentile, median, 75th percentile, and maximum values of the distribution to create 5 representative scenarios for the number of hidden barriers (1875, 3495, 4250, 5200, and 7490, respectively). For unmapped barriers, passability was set to the mean passability of all recorded structures (0.45) in the GMCP database, including those with full passability.

### Approximation of effective river segment length

To calculate expected net habitat ${\tilde{v}}_{j}$ above each barrier, we coded a special Matlab script to estimate effective segment length ${\tilde{l}}_{s}$ on a segment‐by‐segment basis. Given there are 203,281 segments in the Maine river network, this required considerable computational overhead involving several days of calculation. As an approximation, we developed the following log‐linear regression model to derive a multiplier for effective segment length:

(12) $$\begin{array}{ccc} \ln\left(\tilde{l}/l\right) & = & \beta_{1}\left[l_{\mathrm{norm}}\times p\right]+\beta_{2}\left[l_{\mathrm{norm}}\times n\right]+\beta_{3}\left[l_{\mathrm{norm}}^{\prime }\times n\right]\\ & & +\;\beta_{4}\left[l_{\mathrm{norm}}\times p\times n\right]+\beta_{5}\left[l_{\mathrm{norm}}^{\prime }\times p\times n\right], \end{array}$$

where $\tilde{l}/l$ is the ratio of a segment's effective to true length, *l*_norm_ is the normalized length of a segment (i.e., length *l* over total length *L*), $l_{\mathrm{norm}}^{\prime }$ is the normalized river length downstream of a segment (i.e., downstream length l′ over total length *L*), *n* is the total number of hidden barriers, *p* is the mean passability of hidden barriers, and the βs are regression model parameters to be estimated via ordinary least squares (OLS). With OLS parameter estimates in hand, one can compute a multiplier $\theta_{s}=\exp\left(\beta^{T}y_{s}\right)$ for each segment *s* (***β*** being the vector of regression parameters and ***y***_*s*_ the vector of covariates for segment *s*) to approximate effective segment length ${\hat{l}}_{s}=\theta_{s}\cdot l_{s}$.

We estimated model parameters (Equation 12) by taking a sample of 2035 segments from the Maine river network, systematically varying hidden barrier passability (*p* = 0.1, 0.3, 0.5, 0.7, 0.9) and the number of hidden barriers (*n* = 6.25%, 12.5%, 25%, 50%, 100% of the total number of known barriers), and then calculating effective segment length exactly via Equation (11) to produce 50,875 observations. Our approximation model for effective length (Table 1) produced extremely accurate results, with all predictor variables significant to the 0.01 level or better; a pseudo *R* ^2^, measured as the square of the correlation between true and approximated effective length (Eisenhauer, 2003), near 1; a mean absolute error of 1.06 × 10^−4^ (∼0.01%); and a maximum absolute error of 4.42 × 10^−3^ (<1%). Although the model parameter values reported here (Table 1) only apply to Maine, the basic approach for approximating effective segment length can be readily adapted to other locations.

**TABLE 1:** Summary of regression model (Equation 12) results for predicting effective segment length multipliers in Maine watersheds

Parameter	Estimate	SE
β_1_	−11.899*	4.432
β_2_	−0.465**	4.477 × 10^−4^
β_3_	−1.001**	2.251 × 10^−6^
β_4_	0.459**	8.621 × 10^−4^
β_5_	1.001**	3.918 × 10^−6^
Pseudo *R* ^2^	∼1.000	

* *p* ≤ 0.01

** *p* ≤ 0.001.

## RESULTS

**FIGURE 2:**
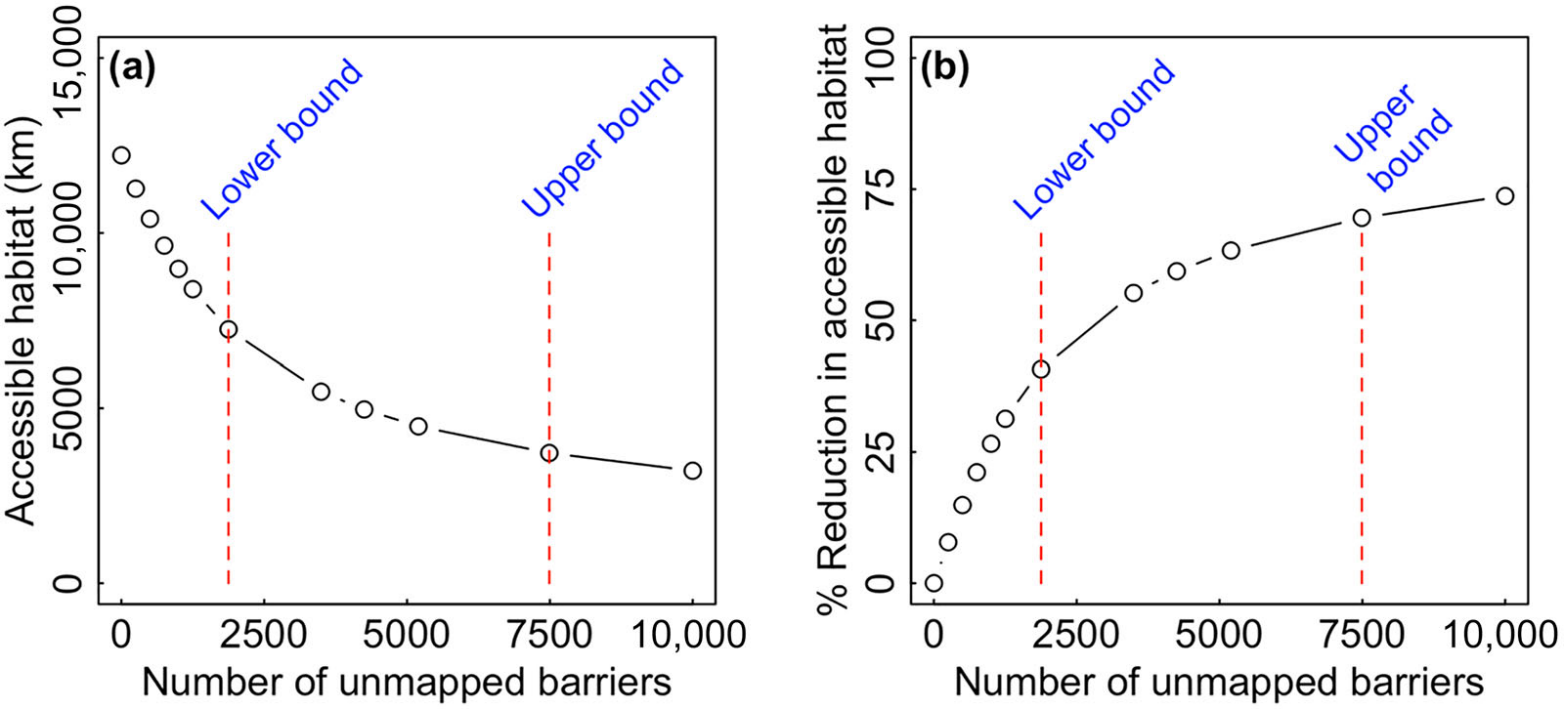
(a) Total accessible habitat of Maine coastal rivers as a function of the number of unmapped barriers and (b) percent reduction in accessible habitat due to unmapped barriers relative to a baseline scenario of no unmapped barriers (dashed red lines, estimated lower and upper bounds on the true number of unmapped barriers).

Failing to acknowledge unmapped barriers led to a dramatic overestimate of current ecosystem connectivity. Under the assumption that no barriers are unmapped, we calculated that only 16.1% of coastal rivers (75,781 km in length) are currently accessible to migratory fishes (Figure 1c). However, the presence of just 1875 unmapped barriers (lower bound estimate) reduced currently accessible habitat by 41% (Figure 2a,b). With 4250 hidden barriers (median estimate), accessible habitat dropped by almost two‐thirds (Figures 1d & 2a,b).

**FIGURE 3:**
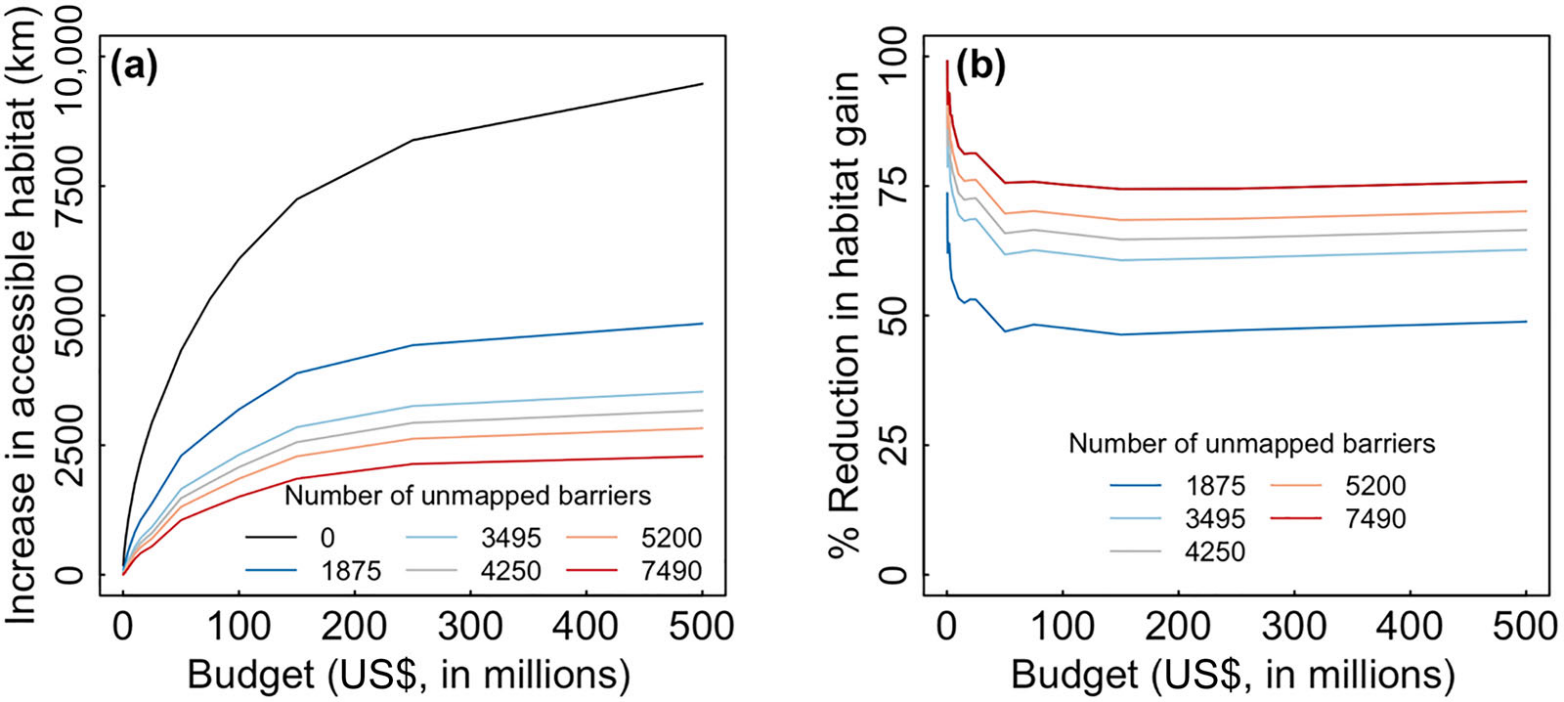
(a) Increase in total accessible habitat in Maine rivers that could be achieved for a given budget when barrier mitigation projects are selected using the naïve model and (b) percent reduction in habitat gain resulting from a given number of unmapped barriers compared with a baseline scenario of no unmapped barriers.

Unmapped barriers also dramatically reduced gains in accessible habitat achieved from barrier mitigation (Figure 3a). For a budget of $100 million, for example, the naïve optimization model identified a set of barrier mitigation projects that would result in a hypothetical increase of 6095 km of accessible habitat. However, this 6095‐km gain was achievable only when no unmapped barriers were present. If there were just 1875 unmapped barriers, barrier mitigation projects identified by the naïve optimization model resulted in only a 3191‐km increase in accessible habitat. Thus, expected habitat gains were 48% lower than expected due to the presence of unmapped barriers (Figure 3b). For the same budget with 7490 unmapped barriers (upper bound estimate), expected habitat gains were fully 75% lower than expected.

**FIGURE 4:**
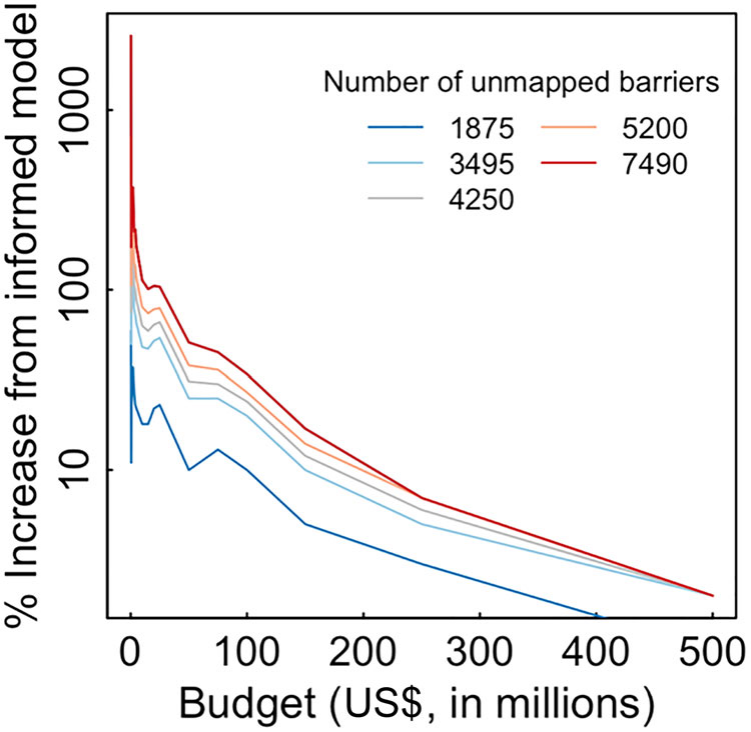
Percent increase in habitat gain that could be achieved by selecting river barrier mitigation projects with the informed model rather than the naïve model. The *y*‐axis is computed as $\frac{\Delta H_{i}-\;\Delta H_{n}}{\Delta H_{n}}\times \;100$, where, for any given budget, $\Delta H_{i}$ is the habitat gain for the informed model solution and $\Delta H_{n}$ is the habitat gain for the naïve model solution taking into account unmapped barriers post facto.

Results for our informed optimization model demonstrated how accounting for missing data during project selection can substantially boost ROI from barrier mitigation (Figure 4). This effect was greatest when budgets were low and the number of unmapped barriers was large. In the scenario with 7490 unmapped barriers, a budget of $2 million resulted in a 218‐km increase in accessible habitat with the informed model, compared with a 46‐km increase if funds were allocated based on the solution to the naïve model. In this case, explicitly accounting for unmapped barriers during project selection increased conservation ROI by 371%. Given 7490 unmapped barriers and budget of $250,000, ROI was a staggering 2586% (26 times) greater for the informed model compared with the naïve model (42 vs. 1.6 km). And although the benefits of an informed approach trended downward as budgets became larger (fewer remaining barriers) or there were fewer unmapped barriers (less uncertainty), additional habitat gains were nonetheless substantial (Figure 4). Averaged over all budgets, anticipating for the presence of hidden barriers increased habitat gain 20–273%, depending on the number of hidden barriers.

**FIGURE 5:**
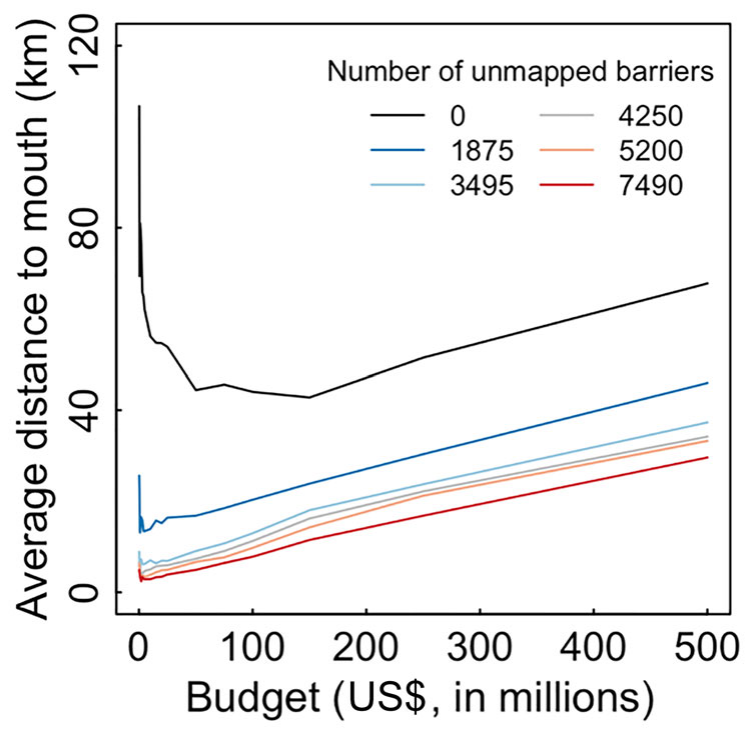
Average distance to river mouth of barrier mitigation projects selected using the informed model.

Accounting for missing data during project selection changed the spatial distribution of barrier mitigation projects (Figure 5). For a budget of $10 million, for example, projects selected using the naïve model were on average 56 km from the mouth of the river. For the same budget and assuming 1875 unmapped barriers, projects selected using the informed model were on average 14 km from the mouth of the river, one‐fourth as far inland. This substantial change in the location of barrier mitigation projects occurred because each unmapped barrier decreased the cumulative passability of all upstream barriers. Thus, when unmapped barriers were present, potential habitat gains were highest for barriers with few potential unmapped barriers downstream (i.e., at mapped barriers close to the river mouth).

**FIGURE 6:**
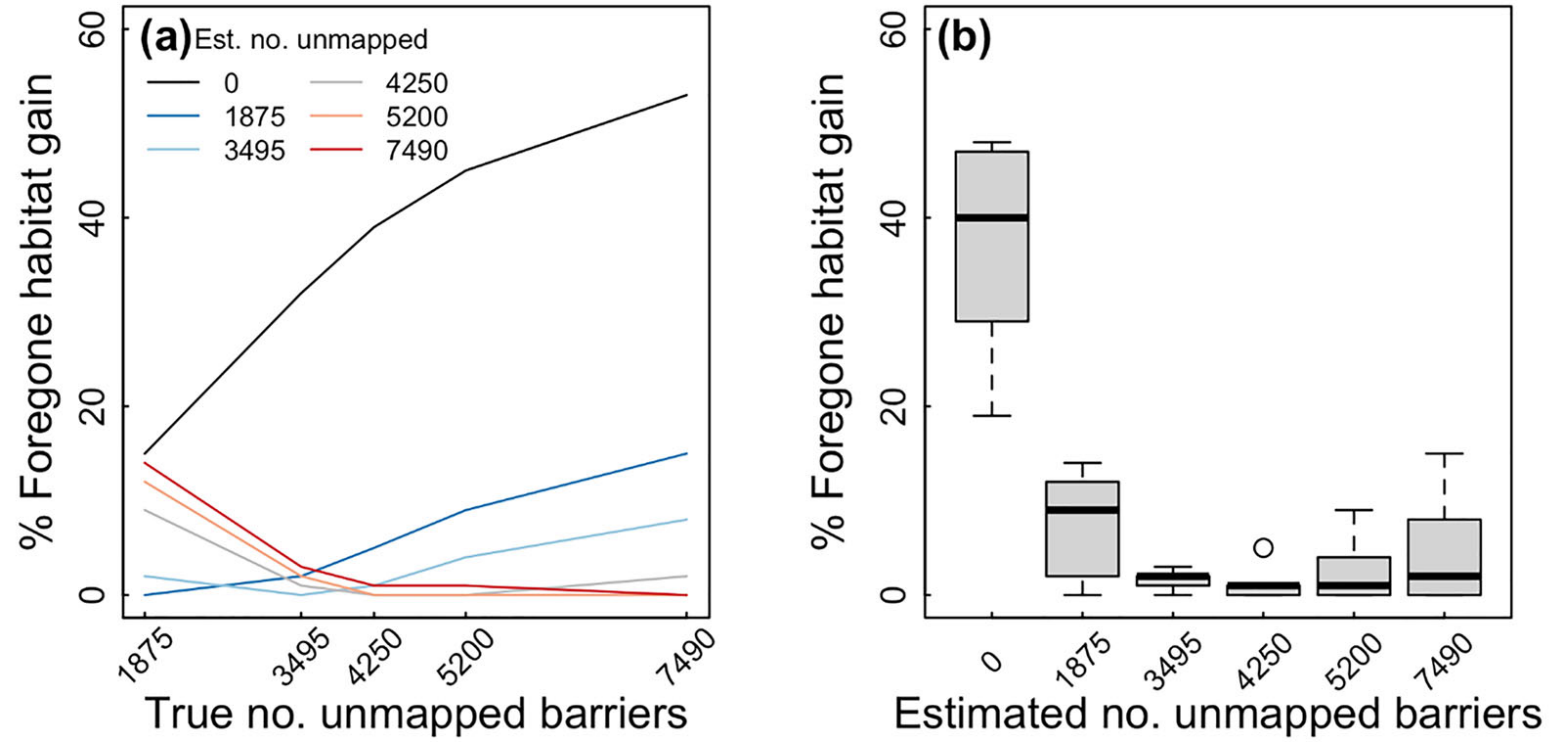
(a) Foregone habitat gain resulting from a mismatch between the true number of unmapped barriers (horizontal axis) and the estimated number of unmapped barriers used to parameterize the informed model and (b) range of potential foregone habitat gain that might occur for an assumed number of unmapped barriers. The true number of unmapped barriers is unknown but assumed to be between 1875 and 7490. Both panels were calculated with a budget of US$10 million.

Given the true number of unmapped barriers is unknown, we analyzed how well solutions optimized for a specific number of hidden barriers might perform when the actual number of hidden barriers differed. Erring too low or too high for the number of unmapped barriers led to significant variability in foregone habitat gain (Figure 6). More precisely, when we assumed a very small or large number of unmapped barriers, little or no foregone habitat gain was forfeited only when the true number of unmapped barriers was comparable. If the true number of hidden barriers deviated considerably, foregone habitat gain was comparatively high. In the most extreme case, assuming no unmapped barriers were present, foregone habitat gain was 53% given a modest $10 million budget. In contrast, the most robust solutions were obtained when we assumed an intermediate number of hidden barriers (4250). Under this assumption, foregone habitat gains were the lowest on average and never exceeded 9% regardless of the true number of unmapped barriers (Figure 6b).

## DISCUSSION

There is growing international interest in dam removals and road crossing upgrades as a means of restoring river connectivity and biodiversity. Our informed optimization model adds to the growing literature on barrier prioritization approaches (Garcia de Leaniz & O'Hanley, 2022) by providing a method for boosting conservation outcomes despite incomplete barrier inventories (Belletti et al., 2020). Overall, our results showed that accounting for unmapped barriers is essential for maximizing river connectivity gains. Critically, improved conservation outcomes can be achieved simply by acknowledging that hidden barriers may be present without even knowing where they are. Moreover, the importance of accounting for unmapped barriers has direct relevance to river conservation practitioners. In particular, as the number of unmapped barriers increased, selection of barrier mitigation projects was directed near to the river mouth.

More broadly, our study demonstrated how ROI frameworks might be modified to explicitly account for incomplete data. For many conservation decisions, the threat of extinctions and extirpations precludes inaction while more data are acquired (Grantham et al., 2009), which means decisions must be made with incomplete data. Furthermore, various studies have shown that it is often not cost‐effective to acquire more data on species’ distributions, habitats, or threats (Grantham et al., 2008; Hermoso et al., 2013). In the absence of additional data, conservation decisions rely on spatial models of species’ distributions (Wilson et al., 2005) or species’ indicators (Fitzpatrick et al., 2021), habitats (Terrado et al., 2016), and threats (Vörösmarty et al., 2010). The effects of these modeled or proxy data on spatial conservation priorities and efficiency are well quantified (Kujala et al., 2018; La Marca et al., 2019; Wilson et al., 2005), but planning algorithms in spatial conservation planning tools like Marxan (Ball & Possingham, 2000), Zonation (Lehtomäki & Moilanen, 2013), and C‐Plan (Pressey et al., 2009) do not explicitly account for incomplete data. In contrast, our results demonstrated how modifying the spatial planning algorithm itself can improve the ROI of conservation projects.

We focused on anadromous fish, but future work could extend our modeling approach to stream‐resident fish and other aquatic organisms (Cote et al., 2009; O'Hanley, 2011; O'Hanley, Wright, et al., 2013), fish population dynamics (Ioannidou & O'Hanley, 2019; Paulsen & Wernstedt, 1995; Ziv et al., 2012), or spatial dynamics (Fitzpatrick & Neeson, 2018). Enhanced prediction of the actual number of unmapped barriers could also be incorporated into our framework and would greatly improve the effectiveness of barrier prioritization decisions. Ramos (1999) suggests the use of Bayesian models to simulate undercount data, whereas Fader and Hardie (2000) propose the use of the beta‐binomial or negative binomial distribution.

Dams and other structures provide many societal benefits (Doyle & Havlick, 2009) and deliberations to remove them inevitably involve balancing a diverse set of costs (e.g., reductions in water provisioning, recreation, flood control, and hydropower generation) and benefits (e.g., ecosystem connectivity improvements and reduced dam failure risk). Our informed optimization model could be extended to consider multiple objectives, including dam safety (Zheng & Hobbs, 2013), water storage and hydropower production (Kuby et al., 2005), recreation (Roy et al., 2018), potential threats from invasive species (Milt et al., 2018), and climate uncertainty (Farzaneh et al., 2021). In Maine, hydropower losses from dam removal could be potentially offset by solar production on a modest land area (Sharma & Waldman, 2021) or via offsetting opportunities (O'Hanley et al., 2020; Owen & Apse, 2015), whereby lost hydropower is compensated by hydropower installation or upgrade elsewhere.

Our results offer broad lessons for conservation practice. Nearly all conservation decisions are made with missing or incomplete data, but data limitations are rarely considered when allocating conservation resources among candidate projects. We demonstrated that simply acknowledging that data are incomplete—and accounting for this shortcoming during project selection—can boost ROI. Given incomplete data on species’ distributions, habitat availability, and threats in ecosystems worldwide, our results highlight the importance of explicitly accounting for incomplete data in conservation planning.

## Supporting information

Supplementary Appendices
