# Supplementary material for: Boosting large‐scale river connectivity restoration by planning for the presence of unrecorded barriers
Source: Conserv Biol. 2023 May 1;37(3):e14093. doi: 10.1111/cobi.14093 (PMC10962602; doi:10.1111/cobi.14093)
Supplement: Supplementary file 1 — Supplementary Appendices [file COBI-37-0-s001.pdf]

Boosting large-scale river connectivity restoration by planning for the presence of unrecorded barriers

Christina T. Ioannidou   Thomas M. Neeson   Jesse R. O'Hanley

Appendix S1: Example barrier network

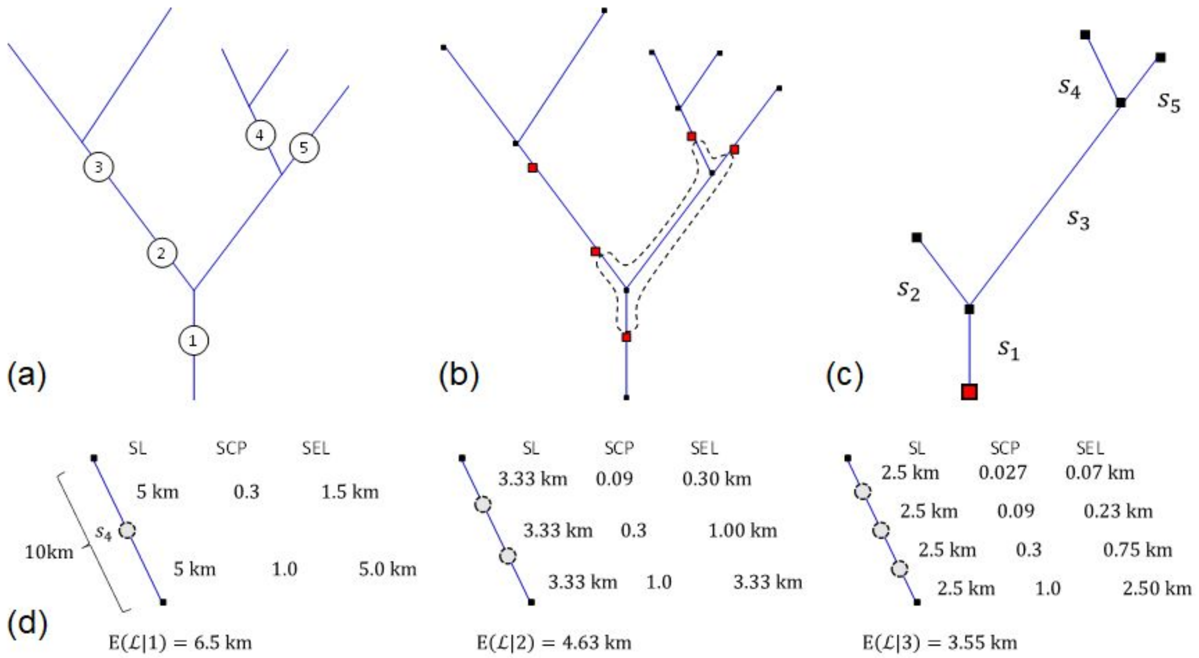

Figure S1: (a) Hypothetical river network with 5 barriers, (b) decomposition of the river network into 14 nonoverlapping river segments (smaller black squares, river confluences and terminuses; red squares, barriers; dashed line, barrier 1's river subnetwork U_1), (c) close-up of the 5 river segments (s_1 to s_5) in river subnetwork U_1, (d) calculation of conditional expected length $\mathrm{E}(\mathcal{L}_s|k)$ according to Eq. 10 (main text) for segment s_4 based on subsegment length (SL) – the $\frac{\ell_s}{k+1}$ in Eq. 10, subsegment cumulative passability (SCP) – the $\tilde{p}^r$ in Eq. 10, and subsegment effective length (SEL) – the product of $\tilde{p}^r$ and $\frac{\ell_s}{k+1}$ in Eq. 10, given k = 1, 2, or 3 hidden barriers (dashed circles), each having expected passability 0.3.

Appendix S2: Extensions to the informed barrier optimization model

In most real-world landscapes, it is often the case that hidden barrier are not randomly located over a river network but instead more likely to be found on smaller sized rivers. To model this, we simply need to modify the event probabilities in π_{skt} (Eq. 8, main text). Let $q(m)$ be the instantaneous probability a hidden barrier

is located on a river segment of size $m = 1, \ldots, M$, with larger values of m indicating larger sized rivers. By presumption $q(m) \geq q(m+1)$ for all $m = 1, \ldots, M-1$. Let o_s be the size of river segment s (i.e., the m value of segment s) and let $w_s = q(o_s) \cdot \ell_s$ be the "weighted" length of river segment s. Further, we define:

w'_s = total weighted length of river downstream from segment s

w''_s = total weighted length of river not downstream of or within segment s

Total weighted length of the river network is donted by $W = \sum_{s \in S} w_s$. With this in place, an alternative expression for probability π_{skt} when the likelihood of hidden barriers being present depends on river segment size (or other characteristics) is given by:

$$\pi_{skt} = \frac{n!}{k!t!(n-k-t)!} \left(\frac{w_s}{W}\right)^k \left(\frac{w_s^{'}}{W}\right)^t \left(\frac{w_s^{''}}{W}\right)^{(n-k-t)} \tag{S1}$$

As an illustration, assume that the probability of hidden barriers being located on river segments is inversely proportional to Strahler stream order, a widely used proxy for river size. This would result in the following specification for probability $q(m)$:

$$q(m) = \frac{\frac{1}{m}}{\sum_{h=1}^{M} \frac{1}{h}} \tag{S2}$$

Applying this to the Maine river network, with a maximum stream order of 8 ($M = 8$), would yield q values of (0.368, 0.184, 0.123, 0.092, 0.074, 0.061, 0.053, 0.046) for river segments of order 1 to 8.

Appendix S3: Proposition and proof of barrier separation distance

Proposition. *Given k hidden barriers located along a river segment of length ℓ, the expected separation distance between barriers is $\frac{\ell}{k+1}$.*

Proof. The position X of each hidden barrier along a river segment is independent and identically distributed (i.i.d.) uniformly on the interval $(0, \ell)$. It is easy to establish (see p. 63 of Gentle, 2009) that the density function of the uniform order statistic $X_{(i)}$, $i = 1, \ldots, k$, is given by:

$$f_{X_{(i)}}(u) = \frac{k!}{(k-i)!(i-1)!} \left(\frac{u}{\ell}\right)^{i-1} \left(1 - \frac{u}{\ell}\right)^{k-i} \text{ for } 0 < u < \ell$$

This corresponds to the density of a Beta random variable with parameters $\alpha = i$ and $\beta = k - i + 1$ on the range $[0, \ell]$ and mean $\mathrm{E}[X_{(i)}] = \dfrac{\alpha}{\alpha + \beta} \cdot \ell = \frac{i\ell}{k+1}$. Accordingly, the average distance between any two adjacent barrier points i and $i+1$, $i = 1, \ldots, k-1$, is evaluated as $\frac{(i+1)\ell}{k+1} - \frac{i\ell}{k+1} = \frac{\ell}{k+1}$. □

Appendix S4: Comparison of the naïve and informed models

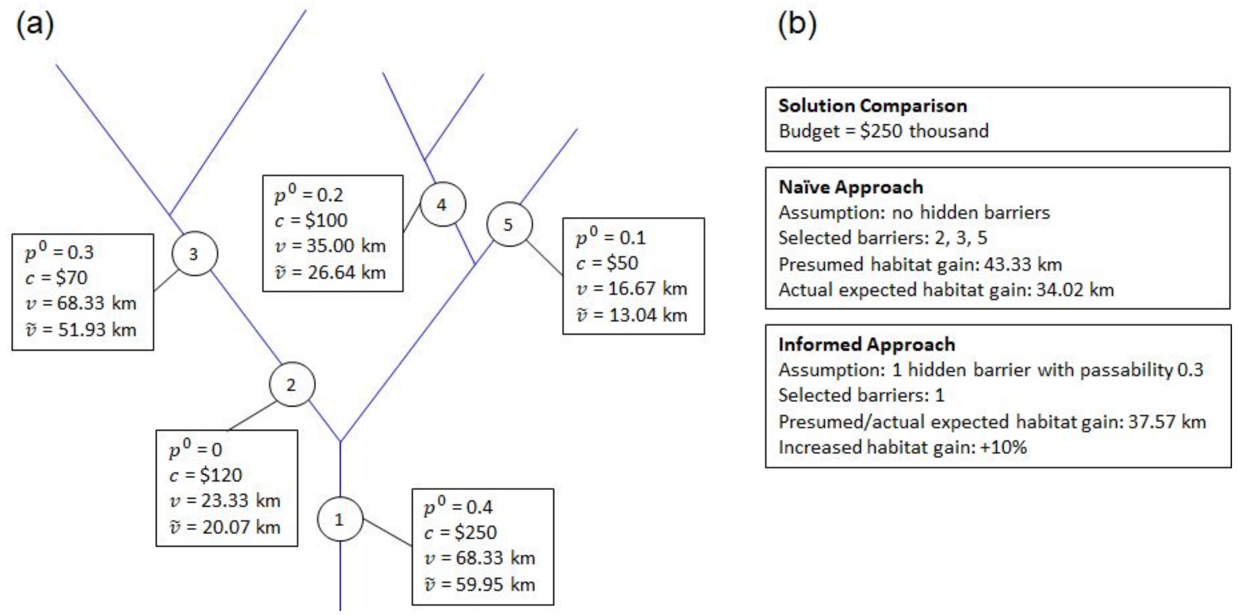

Figure S2: (a) Hypothetical river network with 5 barriers (box contents, basic information about barriers: p^0, current passability; c, cost in thousands of U.S. dollars to increase passability to 1; v, net habitat upstream assuming the barrier inventory is complete; $\tilde{v}$, effective net habitat upstream assuming a single hidden barrier with passability 0.3 is located randomly across the river network) and (b) comparison of naïve and informed models given a budget of US$250 thousand.

Appendix S5: Solution methodology

Effective segment lengths $\tilde{\ell}_s$ can be calculated using the approximation method Eq. 12 (main text) or directly using Eqs. 8-11 (main text) and then aggregated to determine expected net habitat $\tilde{v}_j$ above each barrier using Eq. 7 (main text). In our implementation, we opted for the latter method and subsequently implemented linearized versions of our informed and naïve barrier optimization models (King and O'Hanley, 2016) in C++ using CPLEX callable libraries version 12.9. All experiments were performed on the same dual-core Lenovo ThinkPad T470 laptop (Intel i7-7600U processor, 2.8GHz per chip) with 32 GB of RAM. Solution times varied from under 1 second to 25 seconds, which is remarkable given the large size of the model, which includes 44,706 variables (14,902 binary) and 43,462 constraints.

Appendix S6: Summary of the Maine barrier dataset

Field	Description	Data type	Structure types
SiteID	Barrier ID	Alphanumeric	All
Type	Structure type	Nominal: numerous categories (e.g., dam, culvert, ford, bridge, natural fall)	All
Fishway	Existing fishway type	Nominal: numerous categories (e.g., none, denil, steeppass, pool and weir, lift, rock ramp)	Dams only
Order	Strahler stream order	Ordinal: 1-8	Crossings and dams
BFW	Stream bankfull width (BWF) based on recorded width or Strahler order (if unrecorded)	Numeric: >0	Crossings only
Height	Dam height based on recorded height or Strahler order (if unrecorded)	Numeric: >0	Dams only
Class	Qualitative assessment of current passability	Nominal: barrier, potential barrier, no barrier, unknown	All
Prepass	Est. current passability based on Type, Class, and Fishway (dams only)	Numeric: [0, 1]	All
Cost	Est. mitigation cost based on Type, BWF (crossings only), and Height (dams only)	Numeric: >0	Crossings and dams
Postpass	Est. post-mitigation passability based on Type and Height (dams only)	Numeric: [0, 1]	Crossings and dams

Table S1: Summary of key attributes for river barriers (recorded and estimated) in the Gulf of Maine Coastal Program barrier dataset.

Appendix S7: Supplementary results

Figure S3: Locations of selected barriers with a budget of $10M given 0 (a), 1875 (b), 3495 (c), 4250 (d), 5200 (e), and 7490 (f) hidden barriers.

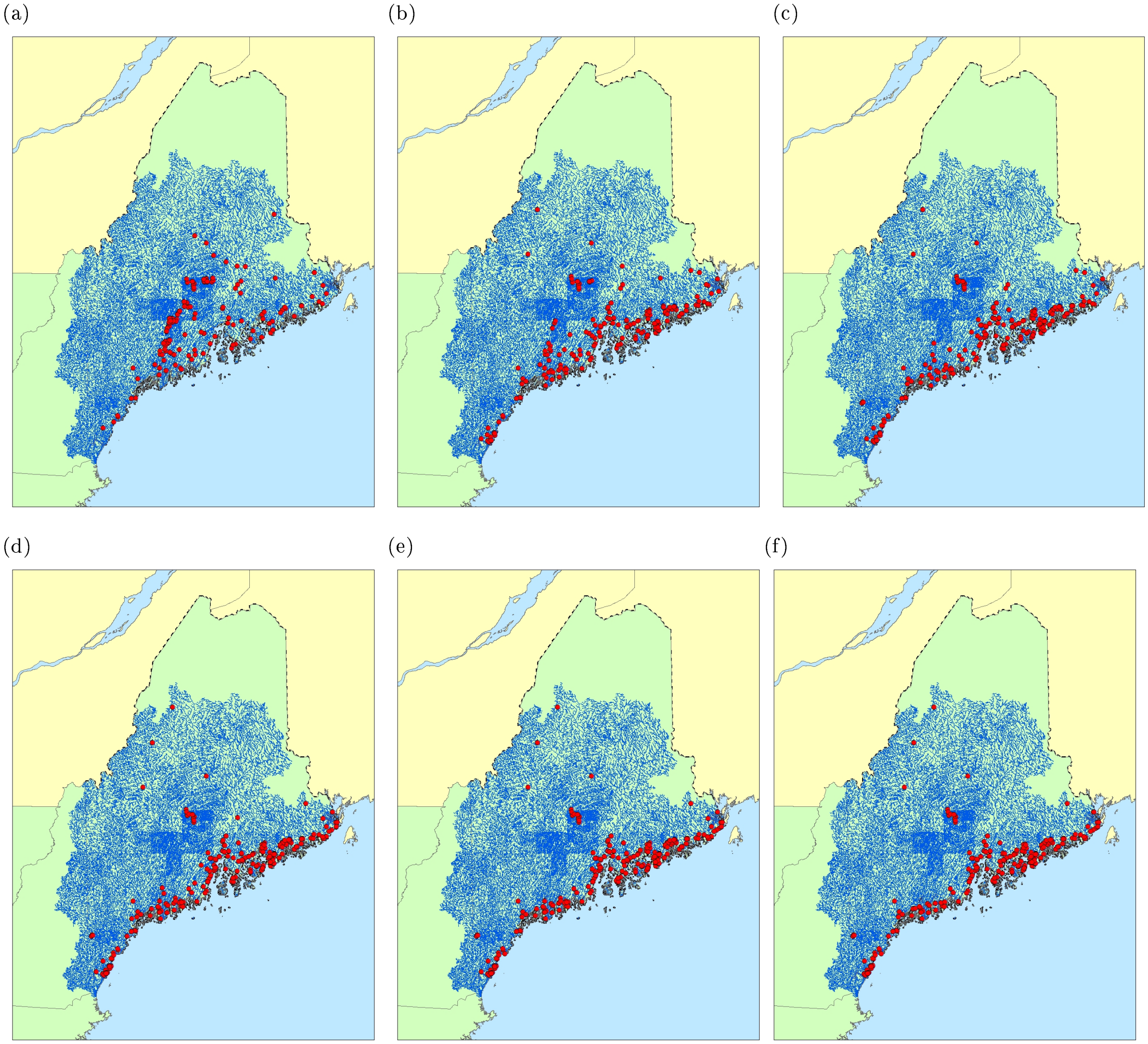

References

Gentle, J.E., 2009. Computational statistics. Springer.

King, S., O'Hanley, J.R., 2016. Optimal fish passage barrier removal - Revisited. River Research and Applications 32, 418–428.
